# Formulations, Hemolytic and Pharmacokinetic Studies on Saikosaponin a and Saikosaponin d Compound Liposomes

**DOI:** 10.3390/molecules20045889

**Published:** 2015-04-03

**Authors:** Guo-Song Zhang, Peng-Yi Hu, Dong-Xun Li, Ming-Zhen He, Xiao-Yong Rao, Xiao-Jian Luo, Yue-Sheng Wang, Yu-Rong Wang

**Affiliations:** 1 School of Chinese Materia Medica, Beijing University of Chinese Medicine, Beijing 100102, China; E-Mails: zhgs81411@aliyun.com (G.-S.Z.); hpy820515@126.com (P.-Y.H.); 2 National Pharmaceutical Engineering Center for Solid Preparation in Chinese Herb Medicine, Jiangxi University of Traditional Chinese Medicine, Nanchang 330004, China; E-Mails: lidongxun0123@aliyun.com (D.-X.L.); hmz07@163.com (M.-Z.H.); rxy1014@163.com (X.-Y.R.); luoxj98@126.com (X.-J.L.); wylw915@163.com (Y.-S.W.)

**Keywords:** saikosaponin a, saikosaponin d, liposomes, formulations, hemolytic, pharmacokinetic

## Abstract

The aim of this study was to develop and optimise a saikosaponin a and saikosaponin d compound liposome (SSa-SSd-Lip) formulation with reduced hemolysis and enhanced bioavailability. A screening experiment was done with Plackett–Burman design, and response surface methodology of five factors (EPC/SSa-SSd ratio, EPC/Chol ratio, water temperature, pH of PBS, and ultrasound time) was employed to optimise the mean diameter, entrapment efficiency of SSa and SSd, and the reduction of hemolysis for SSa-SSd-Lip. Under the optimal process conditions (EPC/SSa-SSd ratio, EPC/Chol ratio, water temperature and pH of PBS were 26.71, 4, 50 °C and 7.4, respectively), the mean diameter, the entrapment efficiency of SSa, the entrapment efficiency of SSd and the hemolysis were 203 nm, 79.87%, 86.19%, 25.16% (SSa/SSd 12.5 mg/mL), respectively. The pharmacokinetic studies showed that the SSa-SSd-Lip had increased circulation time, decreased Cl, and increased AUC, MRT and T_1/2β_ (*p* < 0.05) for both SSa and SSd after intravenous administration in comparison with solution.

## 1. Introduction

**Figure 1:**
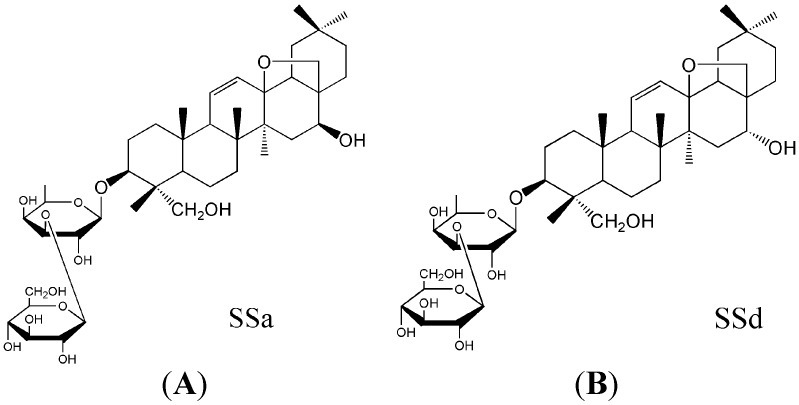
The chemical structure of SSa (**A**) and SSd (**B**).

Saikosaponin a and Saikosaponin d (SSa, SSd, Figure 1) from the prepared radix of *Bupleurum chinense* DC. or *Bupleurum scorzoneri folium* W illd [1], are two active compounds and have many pharmacological activities, including antipyretic, analgesic, antiviral, anti-inflammatory, antitumour, immunomodulatory, liver damage-resistant, and liver fibrosis-resistant properties [2]. Both SSa and SSd play an important role in antifibrosis by improving liver oxidative stress resistance, inhibiting hepatic stellate cell activation, and via anti-inflammatory and anti-hepatitis B virus properties [3]. SSa had stronger anti-inflammatory activity than SSd, which benefits the treatment of inflammation in the late phase of liver fibrosis. However, SSd plays a more important role than SSa in inhibiting hepatic stellate cell activation and anti-hepatitis B virus [4,5,6,7]. SSd in combination with SSa could have obvious synergies effects to treat fibrosis of the liver.

However, SSa and SSd can easily combine with cholesterol of red blood cell walls once injected into the body, leading to insoluble molecular complexes that destroy the permeability of red blood cells and finally produce hemolysis [8]. Embedding drugs in liposomes can reduce drug toxicity and hemolysis [9]. As a biodegradable and biocompatible drug delivery system, liposome has been widely investigated and applied, and is so far the only listed formulation that can be used for intravenous injection of nanodrugs [10]. Liposomes were also reported to greatly reduce the haemolytic effects *in vivo* and *in vitro* for drugs such as amphotericin B, nobiliside A and cucurbitacin B [11,12,13]. Both SSa and SSd are pentacyclic triterpenes and mutual optical isomers, and have similar chemical and physical properties. Because of the stronger lipophilicity, the film dispersion method was used for the preparation of SSa and SSd compound liposomes (SSa-SSd-Lip). There are many process factors that affect the physicochemical and biological properties of SSa-SSd-Lip, such as particle size, entrapment efficiency and hemolysis. Aspects that should be considered are the ratio of phospholipids and drugs, the ratio of phospholipids and cholesterol, pH of the phosphate buffer solution (PBS), water bath temperature and ultrasound time. An HPLC method was developed and validated for the determination of SSa-SSd-Lip thus allowing calculation of their encapsulation efficiencies. The hemolysis of liposomes was evaluated by a UV method [11]. The Plackett-Burman design was used as an efficient tool to screen for significant factors among a large number of variables while minimising the number of experiments [14]. After finding the critical factors, a Box-Behnken design was chosen to optimise the actual values of these process factors combined with a desirability function [15,16].

Liposomes, spherical structures composed of one or several phospholipid bilayers, possess many attractive characteristics to stabilise drugs and to improve their pharmacological properties. When particles are administered intravenously, they are quickly coated by the components of the circulation. Surface hydrophobicity of particles is directly correlated to the kinetics of plasma clearance by the reticuloendothelial system (RES). Liposomes with smaller particle size avoid RES elimination and allow for a longer circulation plasma half-life. In this work, we have studied SSa-SSd-Lip pharmacokinetic profiles after intravenous administration and compared them to a physical mixture of SSa and SSd in order to explore the effect of the formulation.

## 2. Results and Discussion

### 2.1. Hemolysis Test in Japanese Big Ear Rabbit RBCs

**Figure 2:**
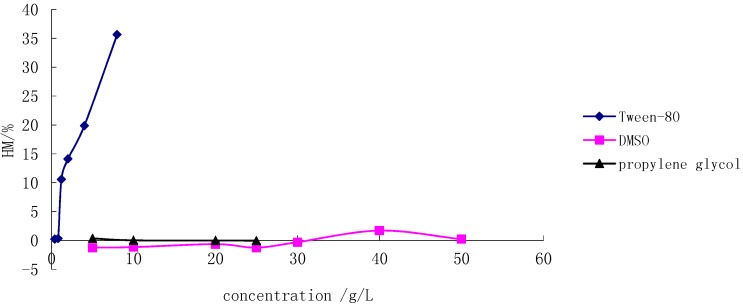
Hemolysis of different co-solvents.

Because of the poor solubility of SSa and SSd in water, Tween-80, DMSO and propylene glycol were selected as co-solvents, and their hemolysis was studied, respectively. Figure 2 shows that Tween-80 led to complete lysis at low concentration, In contrast, DMSO and propylene glycol did not show any hemolysis up to a concentration of 20 g/L. DMSO was finally chosen as a co-solvent since it gave a higher solubility for both SSa and SSd than propylene glycol. The logarithm of drug concentration was as abscissa and the HM% was as the vertical axis. The standard curve equations for SSa, SSd and the physical mixture of SSa and SSd (1:1, g/g) and were Y = 435.42lgC − 218.69, Y = 377.37lgC + 117.93, and Y = 554.19lgC − 5.9279, respectively. The corresponding linear ranges were 3.234–5.544 μg/mL, 0.547–0.860 µg/mL and 1.018–1.488 μg/mL, respectively. Based on the data obtained, both SSa and SSd were considered to be highly haemolytic, and the hemolysis of SSd was even stronger.

UV spectrophotometric methods for testing may be effected by colloidal dispersion, and therefore an emulsion-breaker should be added to decrease the probability of interference. Table 1 shows that a small amount of added 10% X-triton could make the liposome suspension liquid clear, and had less influence on the absorbance. Thus, 10% X-triton was selected as emulsion-breaker. RCB suspension (2.5 mL) was mixed with 2.5 mL of SSa-SSd-Lip, and then 3.0 mL of the suspension resulting from the hemolysis test was collected and added to 0.2 mL of 10% X-triton for analysis of the extent of hemolysis by reading the absorption of the haemoglobin at 575 nm.

**Table 1:** Screening results of the emulsion-breaker.

Demulsifier	The Amount Added (mL)	The Rate of Change of Absorbance (%)
10% X-triton	0.1	0.94
isopropyl alcohol	0.7	61.06
absolute ethyl alcohol	2.0	30.15

### 2.2. Design of Experiments

#### 2.2.1. Screening Study

The experimental results of the Plackett–Burman design are tabulated in Table 2. Twelve experiments were carried out according to the conditions fixed by the experimental design shown in Table 3. ANOVA analysis of the results provided the weights of the experimental factors for all of the response. From these results, it was demonstrated that entrapment efficiency of SSa and SSd, the hemolysis and the mean diameter behaved differently when the process variables were changed. For the EEssa% and EEssd%, the model *F*-value of 33.34 and 45.79, and the *p*-value were 0.0003 and 0.0001, which implied that the two models were significant and indicated that the two fitted models were suitable for use in this experiment. In these cases, the EPC/SSa-SSd ratio (X_1_), water bath temperature (X_3_) and the pH of PBS (X_4_) exhibited significant (*p* < 0.05) effects on the EEssa% and EEssd%. On the other hand, the EPC/Chol ratio (X_2_) and ultrasound time (X_5_) showed little effect (*p* > 0.05) on the yield value of EEssa% and EEssd%. For HM%, the model *F*-value of 18.09 and the *p*-value were 0.0015, which implied that the model was significant and indicated that the model was suitable for use in this experiment. In these cases, only the EPC/Chol ratio (X_2_) showed significant (*p* < 0.05) effects and the other factors exhibited little significance (*p* > 0.05). For mean diameter (MD) and the polydispersity (PDI), the model *F*-value of 1.57 and 3.03, and the *p*-value were 0.2975 and 0.1051, which implied that the two models were not significant and indicated that the two fitted models were not suitable for use in this experiment. In these cases, only the ultrasound time (X_5_) showed significant (*p* < 0.05) effects on PDI, and the other factors exhibited little significance (*p* > 0.05). Table 3 showed that the pH of PBS (X_4_) had effect on the EEssa% and EEssd%, and meanwhile pH7.4 of PBS was fitted, and therefore, we fixed the X_4_ at 7.4 in this study. The ultrasound time (X_5_) did not have much effect on any of the response values except for PDI, and X_5_ at 15 min was chosen on account of the economic perspective. The EPC/SSa-SSd ratio (X_1_), the EPC/Chol ratio (X_2_) and water bath temperature (X_3_) were chosen to further optimize the subsequent Box-Behnken design.

#### 2.2.2. Optimisation Study

The optimisation study was performed using a Box-Behnken design (BBD) in order to find the optimal conditions (Table 4). The EPC/SSa-SSd ratio, the EPC/Chol ratio and water bath temperature were selected for optimisation in the next experimental design, whereas the levels of pH of PBS and ultrasound time were fixed. Table 5 shows the experimental conditions of the BBD along with the corresponding values observed for the three responses studied. Experimental data was fitted to the quadratic model by ANOVA. The ANOVA for the three responses is shown in Table 6. For the EEssa%, EEssd% and HM%, the model *F*-value of 57.88, 26.83 and 150.97, and the *p*-value were both less than 0.0001, which implied that the three models were significant and there was less than 0.01% chance that a “Model *F*-value” this large could occur due to noise. A *p*-value less than 0.05 indicated model terms were significant. In these cases, Table 5 indicated the effects of the EPC/SSa-SSd ratio (X_1_), the EPC/Chol ratio (X_2_) and water bath temperature (X_3_) were all significant model terms with a negative linear relationship with responses. The “Lack of Fit *F*-value” of 3.55, 4.65 and 5.58 implied the “lack of fit” was not significant relative to the pure error. There was a 12.19%, 7.93 and 5.90% chance that a “lack of fit *F*-value” this large could occur due to noise. Non-significant “lack offFit” was good, so we wanted the model to fit, which further validated the model. The coefficients of determination (R^2^) for entrapment efficiency of SSa, entrapment efficiency of SSd and the hemolysis were 0.9860, 0.9415 and 0.9873, respectively, which implied that over 98.60%, 94.15% and 98.73 of the variations for the process efficiency could be explained by these models. The closer *R*^2^ was to 1, the better the empirical models fitted the actual data.

**Table 2:** Process factors and their two levels used in the Plackett-Burman design.

Variable No.	Factors	Unit	Lower Level(-1)	Higher Level(+1)
X_1_	EPC/SSa-SSd	g/g	10	40
X_2_	EPC/Chol	g/g	4	10
X_3_	Water bath temperature	°C	40	70
X_4_	pH of different PBS	-	6.5 ^a^	7.4 ^b^
X_5_	Ultrasound time	min	10	20

^a^ pH6.5PBS: 0.68 g potassium dihydrogen phosphate and 15.2 mL 0.1 mol/L sodium hydroxide solution were diluted to 100 mL by sterilizing water; ^b^ pH7.4PBS: 1.36 g potassium dihydrogen phosphate and 0.79 mL l mol/L sodium hydroxide solution were diluted to 200 mL by sterilizing water.

**Table 3:** Arrangement and results of the Placket-Burman design.

Standard No.		Run		Process Factors								Response Values								
				X1	X2		X3		X4		X5	EE_SSa_%		EE_SSd_%		HM%	MD (nm)			PDI
1		1		1	1		−1		1		1	74.24		77.72		85.99	251			0.246
2		7		1	−1		−1		−1		1	65.87		66.97		48.28	167			0.213
3		6		−1	−1		−1		1		−1	51.79		53.98		0.88	325			0.314
4		12		−1	−1		−1		−1		−1	42.34		47.36		7.26	222			0.271
5		10		−1	1		1		1		−1	45.16		49.76		90.72	411			0.334
6		8		1	1		−1		−1		−1	76.56		76.39		84.19	280			0.417
7		11		1	−1		1		1		1	68.81		67.79		29.78	158			0.231
8		4		−1	1		−1		1		1	53.94		56.28		93.27	228			0.256
9		9		1	1		1		−1		−1	59.32		60.81		90.32	198			0.279
10		2		−1	1		1		−1		1	39.62		40.49		88.93	248			0.246
11		3		1	−1		1		1		−1	70.57		73.15		6.12	294			0.338
12		5		−1	−1		1		−1		1	36.59		40.44		3.31	316			0.226
**Souce**	**EE_SSa_%**					**EE_SSd_%**				**HM%**					**MD (nm)**			**PDI**		
	**F**		***p* -value**			**F**		***p* -value**		**F**			***p* -value**		**F**	***p* -value**		**F**	***p* -value**	
model	33.34		0.0003			45.79		0.0001		18.09			0.0015		1.57	0.2975		3.03	0.1051	
X_1_	139.45		<0.0001			182.92		<0.0001		1.64			0.2475		3.25	0.1216		0.27	0.6233	
X_2_	1.08		0.3378			1.40		0.2816		86.43			<0.0001		0.37	0.5678		1.55	0.2601	
X_3_	13.06		0.0112			21.63		0.0035		0.051			0.8281		0.46	0.5220		0.18	0.6867	
X_4_	12.80		0.0117			21.59		0.0035		0.11			0.7530		1.14	0.3264		0.20	0.6683	
X_5_	0.29		0.6090			1.40		0.2816		2.21			0.1873		2.64	0.1556		12.93	0.0114	

**Table 4:** Factors and levels for the BBD.

Variable No.	Factors	Unit	Lower Level(−1)	Higher Level(+1)
X_1_	**EPC/SSa-SSd**	g/g	10	40
X_2_	**EPC/Chol**	g/g	4	10
X_3_	**Water bath temperature**	°C	40	70
Fixed	**pH of PBS**	-	7.4	
Fixed	**Ultrasound time**	min	15	

**Table 5:** Box-Behnken design and the corresponding response measurements.

Standard No.	Run	Process Factors			Response Values			
		X1	X2	X3	EE_SSa_%	EE_SSd_%	HM%	
1	9	10	4	55	56.85	57.60	35.46	
2	6	40	4	55	77.65	75.20	61.53	
3	1	10	10	55	43.98	48.44	70.33	
4	11	40	10	55	74.70	80.98	92.09	
5	16	10	7	40	54.26	55.73	69.03	
6	3	40	7	40	80.70	81.08	75.94	
7	2	10	7	70	40.61	42.00	42.45	
8	7	40	7	70	50.88	55.68	69.63	
9	10	25	4	40	79.15	83.85	31.2	
10	17	25	10	40	68.43	69.12	58.11	
11	5	25	4	70	50.83	58.23	3.65	
12	15	25	10	70	44.37	47.27	49.33	
13	12	25	7	55	76.46	81.10	38.87	
14	8	25	7	55	81.40	80.57	37.94	
15	4	25	7	55	77.93	79.22	40.8	
16	14	25	7	55	80.13	85.30	41.1	
17	13	25	7	55	79.69	79.03	38.37	

**Table 6:** Analysis of variance (ANOVA) for the regression models.

Response	Source	Sum of Squares	df	Mean Square	*f*-Value	*p*-value	Model
EE_SSa_%	Model	3677.45	7	525.35	57.88	<0.0001	significant
	X_1_	972.84	1	972.84	107.19	<0.0001	
	X_2_	136.20	1	136.20	15.01	0.0038	
	X_3_	1148.71	1	1148.71	126.56	<0.0001	
	X_1_X_3_	65.32	1	65.32	7.20	0.0251	
	X^2^_1_	417.37	1	417.37	45.99	<0.0001	
	X^2^_2_	145.16	1	145.16	15.99	0.0031	
	X^2^_3_	663.62	1	663.62	73.12	<0.0001	
	Residual	81.69	9	9.08			
	Lack of Fit	66.65	5	13.33	3.55	0.1219	not significant
	Pure Error	15.04	4	3.76			
	Cor Total	3759.14	16				
EE_SSd_%	Model	3307.86	6	551.31	26.83	<0.0001	significant
	X_1_	993.77	1	993.77	48.36	<0.0001	
	X_2_	105.70	1	105.7	5.14	0.0467	
	X_3_	937.44	1	937.44	45.62	<0.0001	
	X^2^_1_	485.75	1	485.75	23.64	0.0007	
	X^2^_2_	94.94	1	94.94	4.62	0.0571	
	X^2^_3_	574.32	1	574.32	27.95	0.0004	
	Residual	205.49	10	20.55			
	Lack of Fit	179.73	6	29.95	4.65	0.0793	not significant
	Pure Error	25.76	4	6.44			
	Cor Total	3513.35	16				
HM%	Model	7063.03	6	1177.17	150.97	<0.0001	significant
	X_1_	838.86	1	838.86	107.58	<0.0001	
	X_2_	2381.19	1	2381.19	305.37	<0.0001	
	X_3_	598.93	1	598.93	76.81	<0.0001	
	X_1_X_3_	102.72	1	102.72	13.17	0.0046	
	X_2_X_3_	88.08	1	88.08	11.30	0.0072	
	X^2^_1_	3053.26	1	3053.26	391.56	<0.0001	
	Residual	77.98	10	7.80			
	Lack of Fit	69.65	6	11.61	5.58	0.0590	not significant
	Pure Error	8.32	4	2.08			
	Cor Total	7141.00	16				

#### 2.2.3. Desirability Optimisation

**Figure 3:**
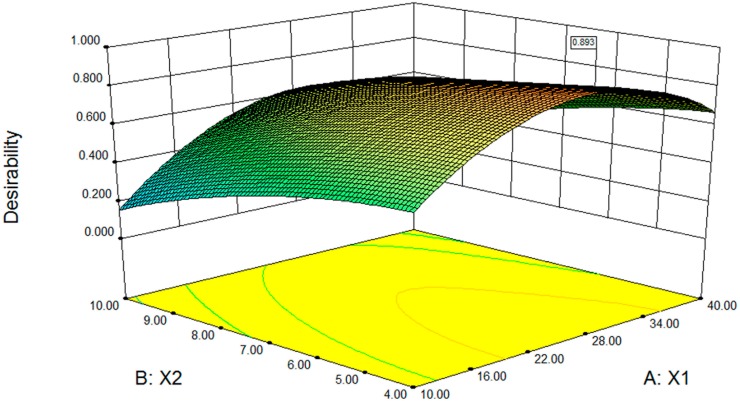
Fitted surface for the desirability as a function of the EPC/SSa-SSd ratio (X_1_) and the EPC/Chol ratio (X_2_). Water bath temperature = 49.96 °C.

The aim of this optimisation was to find the conditions that give the maximum entrapment efficiency and minimum hemolysis. A desirability function approach was used to achieve this goal. Constraints for this optimisation, which were set in the software, can be seen in Table 7. Surface response graphs are presented in Figure 3. The desirability was 0.893. The software predicted that the optimum EPC/SSa-SSd ratio, the EPC/Chol ratio, and the water temperature were 26.71, 4, and 50 °C, respectively. The optimum process were that: (a) SSa and SSd were dissolved in absolute ethanol at 2.5 mg/mL; (b) 400 mg of EPC and 100 mg of cholesterol were dissolved in 10 mL of chloroform and mixed with 3.0 mL of SSa-SSd solution, and then the solvent was slowly removed to form a thin film at 35 °C; (c) the thin film was dispersed in 10 mL of pH 7.4 PBS at 50 °C in a water bath and was broken for 15 min (200 W, intermittent ultrasound 3 s on, 3 s off). The software predicted that the entrapment efficiency of SSa, entrapment efficiency of SSd and the hemolysis were 81.40%, 83.43% and 26.37%, respectively. 

**Table 7:** Constraints of factors and responses for optimization.

Name	Goal	Lower Limit	Upper Limit
the EPC/SSa-SSd ratio (g/g)	Is in a rang	10	40
The EPC/Chol ratio (g/g)	Is in a rang	4	10
the water bath temperature (°C)	Is in a rang	40	70
Entrapment efficiency of SSa (%)	Maximize	40.61	81.40
Entrapment efficiency of SSd (%)	Maximize	42.00	85.30
The hemolysis (%)	Minimize	3.65	92.09

#### 2.2.4. Verification

In order to evaluate the optimisation capability of the models generated according to the results of the Box-Behnken design, the SSa-SSd-Lip were prepared using the optimal process variable settings that X_1_, X_2_ and X_3_ were equal to 26.71, 4, and 50 °C, respectively. The entrapment efficiency of SSa and SSd, and the hemolysis obtained with the predicted models are shown in Table 8. The mean diameter was 203 nm, and the PDI was 0.179. Under the transmission electron microscope, the spherical structures or nearly orbicular in shape were observed. The results showed good agreement of preparation properties with theoretical predictions.

**Table 8:** Model-predicted and observed values of entrapment efficiency of SSa and SSd, the hemolysis according to the optimal experimental conditions (X_1_ = 26.71, X_2_ = 4, X_3_ = 50 °C) (*n* = 3).

Dependent Variable	Predicted Value	Observed Value	Bias */%
EE_SSa/_% (Y_1_)	81.40	79.87	1.88
EE_SSd/_% (Y_2_)	83.43	86.19	−3.31
HM/% (Y_3_)	26.37	25.16	4.59

* Bias was calculated according to equation: Bias/% = (predicted value − observed value)/predicted value × 100%.

### 2.3. Pharmacokinetic Studies of SSa-SSd-Lip

#### 2.3.1. Method Validation

##### Matrix Effect and Recovery

Matrix effect represented as ion suppression for this method. ME was consistent in all of the lots and did not affect the quantitative analysis of analytes and the IS peak. The results are shown in Table 9. The data indicated that no significant matrix effect was observed. The extraction recoveries in rat plasma for SSa and SSd were 92.3%, 93.5%, 92.7% and 91.6%, 95.4%, 93.5% at three levels, respectively (*n* = 6), and that for IS (1000 ng/mL) was 92.9% (*n* = 6). The data indicated that the recoveries of analytes and IS were satisfactory.

**Table 9:** Extraction recovery and internal standard (IS) normalized matrix effect factor of SSa, SSd and Notoginsenoside R_1_ in rat plasma quality control samples (*n* = 6).

Compounds	Nominal Concentration (ng/mL)	Recovery (%)	RSD (%)	IS Normalized Matrix Factor (%)	RSD (%)
SSa	5.00	92.3	12.3	0.97	9.4
	300	93.5	5.7	0.94	8.7
	1500	92.7	6.9	0.92	3.6
SSd	5.00	91.6	13.4	0.96	11.2
	300	95.4	7.2	0.89	8.3
	1500	93.5	8.0	0.93	5.0
IS	1000	92.9	7.7		

##### Linearity, Sensitivity, Carryover and Specificity

Linear calibration curves with coefficients of determination (r^2^) greater than 0.990 were obtained in a concentration range of 3.00–6000 ng/mL in all validation runs. Sensitivity was evaluated by analysing the LLOQ samples (*n* = 6) in all of these three validation runs. LLOQ was defined as the lowest concentration that could be reliably quantitated from the background level. In this case, the LLOQs for SSa and SSd were 1.00 and 0.50 ng/mL, respectively.

The potential carryover effect from the previous concentrated samples could be neglected, since no peaks in the chromatographic region of the analytes of interest were observed by injecting blank plasma extract immediately after the ULOQ sample.

**Figure 4:**
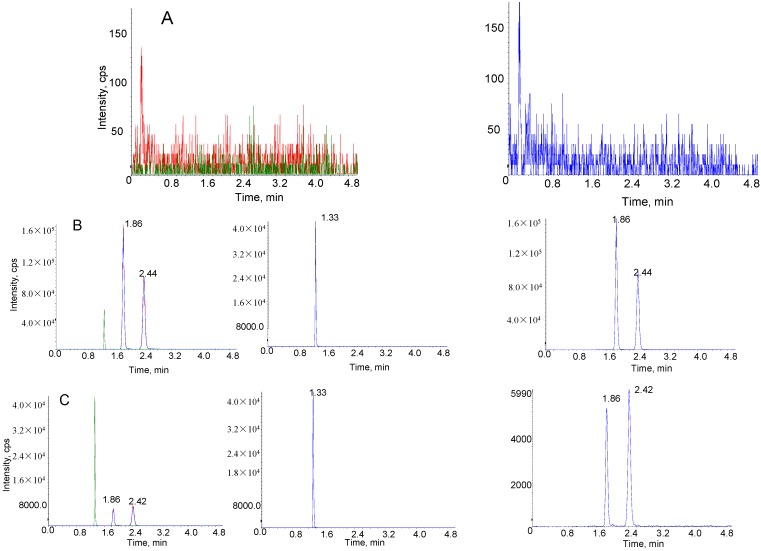
Representative multiple reaction monitoring chromatograms of SSa, SSd and IS in rat plasma. (**A**) Blank plasma sample; (**B**) plasma sample obtained 60 min after intravenous administration of SSa-SSd-lip at dosages of SSa and SSd were 1 mg/kg; (**C**) blank plasma sample spiked with 5.00 ng/mL of SSa/SSd and 1000 ng/mL of IS.

Figure 4 shows the typical multiple reaction monitoring chromatograms of blank rat plasma, a rat plasma sample obtained at 60 min after intravenous administration of SSa-SSd-lip and spiked plasma sample with SSa, SSd and IS. The retention times of SSa, SSd and IS were 1.86, 2.44 and 1.33 min, respectively. There was no significant interference from endogenous substances on the retention time of SSa, SSd and IS.

##### Precision and Accuracy

Precision and accuracy of the assay were determined by replicate analyses (*n* = 5) of four QC levels of 5.00, 300, 1500 and 15,000 ng/mL during the validation. Intra-run and inter-run precision and accuracy for the analysis of SSa and SSd in rat plasma are presented in Table 10 and Table 11, respectively. These show that the LC/MS/MS assay is excellent for the simultaneous quantitative analysis of two analytes in rat plasma.

##### Dilution Integrity

The upper concentration limit of SSa and SSd can be extended to 15,000 ng/mL by a 10-fold dilution with screened rat blank plasma. As shown in Table 10 and Table 11, the results demonstrated that samples with a concentration greater than the upper limit of the calibration curve could be quantified with reliable precision and accuracy after being appropriately diluted with blank matrix.

**Table 10:** Intra-batch accuracy and precision for the analysis of SSa and SSd in rat plasma (*n* = 5).

Statistical Variable	SSa				SSd			
Nominal concentration (ng/mL)	5.00	300	1500	15,000	5.00	300	1500	15,000
Mean(ng/mL)	4.87	307	1469	16,327	4.78	296	1488	15,460
RSD (%)	4.3	5.2	7.1	8.5	5.9	4.6	4.5	7.4
Accuracy (%)	97.4	102.3	97.9	108.8	95.6	98.7	99.2	103.1
RE (%)	−2.6	2.3	−2.1	8.8	−4.4	−1.3	−0.8	3.1

**Table 11:** Inter-batch accuracy and precision for the analysis of SSa and SSd in rat plasma (*n* = 5).

Statistical Variable	SSa				SSd			
Nominal concentration(ng/mL)	5.00	300	1500	15,000	5.00	300	1500	15,000
Mean(ng/mL)	4.91	286	1537	14269	5.14	293	1490	15,540
RSD (%)	5.0	5.3	5.5	7.1	3.6	4.8	5.2	6.9
Accuracy (%)	98.2	95.3	102.5	95.1	102.8	97.7	99.3	103.6
RE (%)	−1.8	−4.7	2.5	−4.9	2.8	−2.3	−0.7	3.6

##### Stability

SSa and SSd were stable in extracted plasma samples for 24 h at room temperature. Both analytes were stable during three freeze/thaw cycles. The SSa and SSd-spiked plasma samples stored at −20 °C were stable for 20 days.

#### 2.3.2. Pharmacokinetic Study

**Figure 5:**
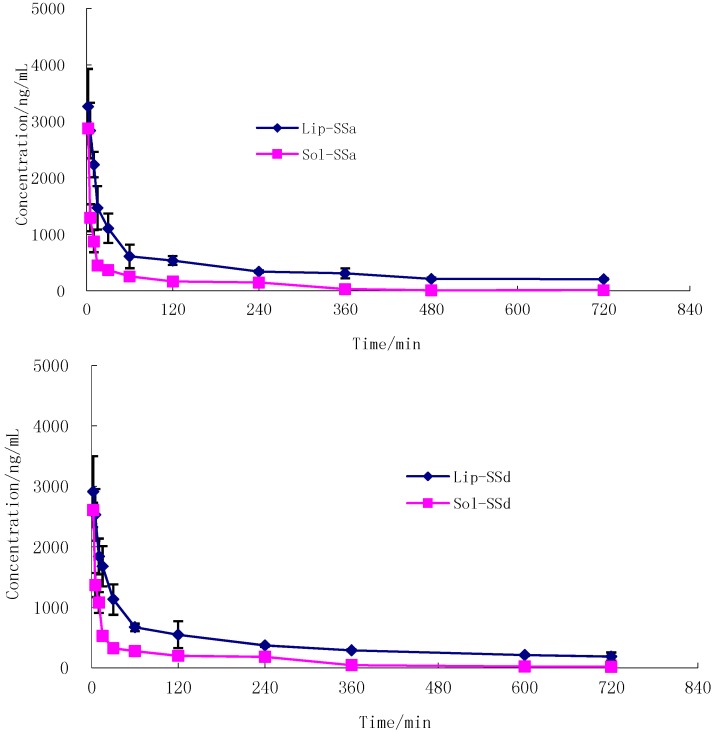
Mean concentration–time profile of SSa (**A**) and SSd (**B**) in rat plasma after a single intravenous administration of SSa-SSd-Lip (SSa 1 mg/kg, SSd 1 mg/kg, *n* = 6) and SSa-SSd-Sol (SSa 1 mg/kg, SSd 1 mg/kg, *n* = 6).

As shown in Figure 5, the decrease in plasma SSa and SSd concentration with time for SSa-SSd-Sol and SSa-SSd-Lip followed a biexponential clearance model. After i.v. administration of liposome formulations to rats, a fall in plasma drug level was found up to 1 h followed by a pattern of slow clearance up to 12 h. The biphasic clearance was more pronounced in this case than with SSa-SSd-Sol. This may be due to the slow release of drug from liposomes and could be explained by the better stability (*in vivo*) achieved after encapsulation in liposomes, and thus reduced the clearance of the drug. The pharmacokinetic parameters derived from the plasma concentration time profile are summarized in Table 12. There was a marked increase in area under SSa and SSd concentration *vs.* time curve of SSa-SSd-Lip; that is, 410.6 μg min/mL for SSa and 384.5 μg min/mL for SSd compared to SSa-SSd-Sol. The AUC of SSa and SSd of SSa-SSd-Sol was significantly lower than SSa-SSd-Lip (90.0 μg min/mL for SSa and 100.4 μg min/mL for SSd). It denotes the slow and steady clearance of the drug when intercalated in liposomes. Elimination half-life (T_1/2__β_) and mean residence time (MRT) were found to be increased for SSa-SSd-Lip, compared with SSa-SSd-Sol. The clearance of SSa-SSd-Sol was 11.3 and 10.0 mL·mg/min for SSa and SSd, and it was 2.4 and 2.6 mL mg/min for SSa-SSd-Lip. Thus increase in AUC and reduction in clearance rate with SSa-SSd-Lip signifies the extended bioavailability of the drug in blood for a longer period of time.

**Table 12:** Pharmacokinetic parameters of SSa and SSd in rats with intravenous administration of SSa-SSd-Lip and SSa-SSd-Sol (mean ± SD, *n* = 6).

Parameter	SSa-SSd-Liposome		SSa-SSd-Solution	
	SSa	SSd	SSa	SSd
T_1/2__β_ (min)	399.8 ± 23.7	346.8 ± 34.1	127.9 ± 22.4	140.3 ± 19.8
MRT (min)	233.9 ± 53.2	228.2 ± 35.9	126.1 ± 17.9	141.5 ± 11.5
Vc (mL/kg)	1404.7 ± 87.2	1301.3 ± 74.8	2050.9 ± 328.2	2015.2 ± 268.0
CL (mL·mg/min)	2.4 ± 0.37	2.6 ± 0.33	11.3 ± 2.58	10.0 ± 3.76
AUC_0__–__t_ (μg·min/mL)	291.0 ± 67.5	290.8 ± 86.9	87.3 ± 20.1	96.7 ± 15.4
AUC_0__–__∞_ (μg·min/mL)	410.6 ± 79.3	384.5 ± 103.4	90.0 ± 18.3	100.4 ± 37.4

## 3. Experimental Section

### 3.1. Materials

Saikosaponin a and Saikosaponin d were obtained from Chengdu Pufeide Biotechnology Co., Ltd (Sichuan, China), purity 98%. Egg phosphatidylcoline (EPC, PC-98T) and cholesterol (Chol) were purchased from AVT pharmaceutical technology Co., Ltd (Shanghai, China). Notoginsenoside R_1_ (IS) was purchased from the National Institute for the Control of Pharmaceutical and Biological Products. Methanol and acetonitrile was of HPLC grade (Merck, Darmstadt, Germany). Ultrapure water used for the LC/MS/MS was from a Milli-Q water purification system (Millipore, Boston, MA, USA). All other chemicals and reagents were of analytical grade and were used as received.

### 3.2. Preparation of Liposomes

According to the weight ratio of 1:1, SSa and SSd were dissolved in absolute ethanol at 2.5 mg/mL. EPC (400 mg) and moderate Chol were dissolved in 10 mL of chloroform and mixed with SSa and SSd solution. The solvent was slowly removed to form a thin film at 35 °C using a B-490BvChl rotary evaporation instrument, and finally the thin film was dispersed in PBS at a predetermined temperature in a water bath and was broken to reduce the particle size by a JY92-IIN ultrasonic cell crusher (200 W, ultrasound 3S, interval 3S). 

### 3.3. Hemolysis Assay

Venous blood was obtained from a Japanese big ear rabbit (3 kg, male) supplied by Longpin Rabbits Breeding Co., Ltd (Nanchang, China). Blood was treated by apheresis and red blood cells (RBCs) were stored at 4 ± 2 °C. Whole blood was centrifuged (15 min at 362 g), and the supernatant were pipetted off and discarded. RBCs were then washed twice with normal saline and were finely dispersed in it at cell densities of 2% (v/v), then stored at 4 °C. Each sample (2.5 mL) was mixed with 2.5 mL of the RCB suspension, and was then incubated at 37 °C in a water bath. After 3 h of incubation, hemolysis was stopped by reducing the temperature to 0 °C), and unlysed RBCs were removed by centrifugation (15 min at 362 g). The supernatants were collected for analysis of the extent of hemolysis by reading the absorption of the haemoglobin. The supernatants were collected and emulsion-breaker was added for analysis of the extent of hemolysis by reading the absorption of the haemoglobin at 575 nm. Results from triplicate experiments are expressed as the percentage of hemolysis with respect to the amount of haemoglobin released in the presence of water, which was taken as the measure of complete (100%) lysis.

(1) $$Hemolysis(\%)=\frac{100({\text{Abs}}_{s}-{\text{Abs}}_{b})}{{\text{Abs}}_{1}-{\text{Abs}}_{b}}$$

where Abs_s_ is the absorbance of the sample, Abs_b_ is the average absorbance of normal saline, and Abs_1_ is the average absorbance of the lysed samples.

#### 3.3.1. The Choice of Co-Solvent

Tween-80 (0.1, 0.2, 0.3, 0.5, 1.0 and 2.0 g) was accurately weighted and added to a 250-mL volumetric flask containing normal saline. Propylene glycol (0.5, 1.0, 2.0 and 2.5 g) was accurately weighted and added to a 100-mL volumetric flask containing normal saline. DMSO (0.5, 1.0, 2.0, 2.5, 3.0, 4.0 and 5.0 g) was accurately weighted and added to a 100-mL volumetric flask containing normal saline. The hemolysis test of each sample was determined according to the above method.

#### 3.3.2. The Choice of Emulsion-Breaker

SSa-SSd-Lip was dispersed in normal saline at a final concentration of 50 µg/mL (SSa/SSd 25 µg/mL), and then 5 mL of the diluted liposome was added. Different emulsion-breakers, including absolute ethyl alcohol, 10% X-triton and isopropyl alcohol, and the quantities of emulsion-breaker that made a visually clear solution, were recorded. RCB suspension (2.5 mL) was mixed with 2.5 mL of sterile water, and then incubated at 37 °C in a water bath. After 3 h of incubation, hemolysis was stopped by reducing the temperature to 0 °C, and unlysed RBCs were removed by centrifugation (15 min at 362 g). Supernatants (3.0 mL) were added to appropriate quantities of different emulsion-breakers and were analysed by reading the absorption of the haemoglobin at 575 nm. Results from triplicate experiments are expressed as the rate of changes of absorbance.

(2) $$Rate(\%)=\frac{100({\text{Abs}}_{a}-{\text{Abs}}_{b})}{{\text{Abs}}_{b}}$$

where Abs_b_ is the absorbance of supernatants before adding the emulsion-breaker, while Abs_a_ is the absorbance of supernatants after adding the emulsion-breaker. The solvent that produced the lowest rate (%) was selected as the emulsion-breaker.

### 3.4. Determination of Entrapment Efficiency (EE%)

The contents of SSa and SSd in compound liposome were determined using an Agilent 1200 HPLC system (Agilent, Palo Alto, CA, USA). Approximately 0.2 mL of SSa-SSd-Lip was dissolved in 10 mL of methanol, and a 20-µL aliquot of the resulting solution was injected into the HPLC system, using a Hypersil ODS C18 column (250 × 4.6 mm, 5 μm) at 25 °C. The mobile phase was a mixture of acetonitrile and H_2_O (45:55, v/v). The flow rate was 1.0 mL/min. Effluent was monitored at 210 nm.

SSa-SSd-Lip (0.2 mL) was sampled to an autonomous sephadex G-50 microcolumn and centrifuged for 2 min (at 1450 g), and the centrifugate was collected. After that, 0.2 mL of PBS was added to the microcolumn using the same conditions, and the centrifugate was collected and combined three times. Merged centrifugate was made up to 10 mL with methanol. The SSa and SSd were analysed by HPLC for the incorporated drug concentration to determine the entrapment percentage. Methanol (0.8 mL) was added to the microcolumn to elute the unincorporated drugs seven times.

The concentrations of SSa and SSd in the compound liposome (n_1_) and the incorporated drugs (n_2_) were assayed by HPLC after dilution with methanol. EE% could be determined by the following equation: EE% = n_2_/n_1_ × 100%. 

### 3.5. Particle Size

The mean diameter (MD) and the polydispersity (PDI) were analysed using a Zetasizer Narcos (Malvern, Malvern, UK).

### 3.6. Design of Experiments

#### 3.6.1. Screening Study

These experiments were designed and analysed using Design-Expert 8.0.5. Experiments were performed in randomised order according to the run number arranged by the software. Five experimental factors were investigated through a design matrix of 12 experiments using a simple linear model (Equation (3)):

(3) $$\text{Y}={\text{β}}_{0}+\sum_{\text{i}}{\text{β}}_{i}{\text{X}}_{i}$$

where Y is the dependent variable, β_i_ represents the parameter estimates, X_i_ is the level of the independent variables, and β_0_ is the model constant. The experimental response is expressed in terms of the entrapment efficiency of SSa (Y_1_, %), the entrapment efficiency of SSd (Y_2_, %), the hemolysis (Y_3_, %), the mean diameter (Y_4_, nm), and the polydispersity (Y_5_). The parameters were as follows: (1) the EPC/SSa-SSd ratio (X_1_, g/g); (2) the EPC/Chol ratio (X_2_, g/g); (3) water bath temperature (X_3_, °C); (4) pH of PBS(X_4_); and (5) ultrasound time(X_5_, min). The extreme levels of each factor were set based on preliminary experiments and the literature. The response values were the mean of three duplicate measurements. Analysis of variance (ANOVA) was used to estimate the significance of main effects and interactions. Factors with a negligible effect on the response at a significance level of 95% were screened out. The remaining factors that affected the response were optimised further.

#### 3.6.2. Optimisation Study

According to the principal of Box–Behnken design, the EPC/SSa-SSd ratio (X_1_, g/g), the EPC/Chol ratio (X_2_, g/g), and water bath temperature (X_3_, °C) were defined as independent values and evaluated on four response values through a design matrix of 17 experiments using a quadratic model (Equation (4)). The entrapment efficiency of SSa (Y_1_, %), the entrapment efficiency of SSd (Y_2_, %) and the hemolysis (Y_3_, %) were defined as the response values in the mathematical modelling. Each of the 17 formulations of a trial was produced three times.

(4) $$\text{Y}={\text{β}}_{0}+\sum_{\text{i}}{\text{β}}_{i}{\text{X}}_{i}+\sum_{\text{i}>\text{j}}{\text{β}}_{\mathrm{ij}}{\text{X}}_{i}{\text{X}}_{j}+\sum_{\text{i}>\text{j}}{\text{β}}_{\mathrm{ij}}{{\text{X}}_{i}}^{2}$$

where Y is the dependent variable, β_i_, β_ij_, β_ii_ are the parameter estimates, X_i,j_ are the levels of the independent variables and β_0_ is the model constant.

### 3.7. Pharmacokinetic Studies of SSa-SSd-Lip

#### 3.7.1. Chromatographic Conditions

An HPLC system consisting of a solvent-delivery system LC-30 AD, an autosampler SIL-30 AC, a column oven CTO-30 AC, a solvent degasser DGU-20A3 and a controller CBM-20A from Shimadzu (Kyoto, Japan) was used in the study. Separation was conducted using a Waters SunFire C_18_ column (50 × 2.1 mm, 5 μm; Milford, CT, USA). The column oven was maintained at 20 °C. The mobile phase was water as mobile phase A and acetonitrile as mobile phase B. The linear gradient elution program was set according to preliminary tests: 10% B, 0–0.6 min; 10%–40% B, 0.6–0.8 min; 40%–60% B, 0.8–3.8 min; 60%–90% B, 3.8–4.0 min; 90% B, 4.0–4.5 min; and 10% B, 4.5–5.0 min, with the flow rate kept at 0.6 mL/min. The injection volume was set at 3 μL.

#### 3.7.2. Mass Spectrometric Conditions

The MS analysis was performed on a 4500 QTRAP^TM^ system from Applied Biosystems (MDS-Sciex, Concord, ON, Canada) equipped with Turbo V sources and TurboIonspray^TM^ interface. Electrospray ionization was performed in negative mode. The mass spectrometric parameters were optimised as: Turbo Ion Spray (TIS) temperature, 650 °C; ion spray voltage, −4500 V; curtain gas, nitrogen, 30; nebulizing gas, 50; TIS gas, 50; declustering potential, −180 V for SSa, SSd and IS; entrance potential, −10 V; collision energy, −50 eV for SSa, −51 eV for SSd and −54 eV for IS; collision cell exit potential, −10 V. The precursor-product ion airs used in multiple reactions monitoring mode were 779.4–617.4 for SSa, 779.4–617.3 for SSd and 931.7–637.1 for notoginsenoside R_1_ with dwell times of 100 ms; quadrupoles Q_1_ and Q_3_ were set on unit resolution. Analyst Software^TM^ (version 1.6.1) was used to process the obtained data.

#### 3.7.3. Preparation of Stock and Working Solutions, Calibration Standards and Quality Control Samples

Standard stock solutions of SSa/SSd (SSa 30.0 μg/mL, SSd 30.0 μg/mL) and notoginsenoside R_1_ (10.0 μg/mL) were prepared in methanol. Working solutions of SSa/SSd at the desired concentration for the preparation of calibration standards and quality control (QC) samples were made daily by serial dilution with methanol. All neat solutions were stored at −20 °C for a maximum of 3 months. Calibration standards were prepared daily by spiking appropriate amounts of stock and working solutions into drug-free rat plasma samples with final concentration levels of 2.00, 5.00, 10.0, 50.0, 100, 200, 1000 and 2000 ng/mL. Similarly, QC samples were prepared at concentrations of 5.00 (low QC, LQC), 300 (medium QC, MQC), 1500 (high QC, HQC) and 15,000 ng/mL (diluted QC, DQC). All QC samples were aliquoted into 2.0-mL centrifuge tubes and stored at −40 °C.

#### 3.7.4. Sample Preparation

Male Sprague–Dawley rats weighing 180–200 g were obtained from the Jiangpu Animal Breeding Center (Nanjing, China), and the studies were approved by The Institutional Animal Care and Use Committee at Jiangxi University of Traditional Chinese Medicine. Animals were housed under standard conditions of temperature, humidity and light. Food and water were freely available. Rats were fasted overnight before the day of the experiment. The developed LC-MS/MS method was applied to investigate the pharmacokinetic profile of SSa and SSd after intravenous administration of SSa-SSd-lip and SSa-SSd-solution (SSa-SSd-sol). The dosages of SSa and SSd were 1 mg/kg. Rats were randomised into two groups for liposome and solution treatment, each group consisting of six rats. Blood samples (0.2 mL) were collected at 2, 5, 10, 15, 30, 60, 120, 240, 360, 480 and 720 min post-dose after intravenous administration. Plasma was collected after centrifugation and stored at −40 °C until analysis. 

A simple and rapid protein precipitation method was used for the preparation of plasma samples. After thawing to room temperature, 50 μL of plasma samples was mixed with 430 μL methanol, and then 20 μL IS solution at 5.0 μg/mL was added. Calibration standards, QC samples, incurred samples and blank matrix control samples were mixed, respectively, by vortex agitation for 3 min and then centrifuged for 10 min at 10,000 rpm. The supernatants were transferred to autosampler vials and injected into the LC-MS/MS system. The SSa and SSd concentrations in rat plasma were calculated using standard curves. WinNolin 5.2 was used to analyse the pharmacokinetic parameters of the area under the plasma concentration–time curve (AUC_0–__t_), the apparent volume of distribution (Vc), total body clearance (CL), elimination half-life (T_1/2__β_) and mean residence time (MRT) of SSa and SSd for each group.

#### 3.7.5. Bioanalytical Method Validation

The validation of the above method was carried out in accordance with US Food and Drug Administration (2013) draft guidelines. The parameters determined were matrix effect, recovery, specificity, sensitivity, carryover, linearity, precision, accuracy, dilution integrity, stability and sample collection stability. 

The matrix effect was determined by comparing the peak areas of SSa, SSd and IS spiked in protein-precipitated blank plasma samples with those of analytes in neat solution at equivalent concentrations. The matrix effect of the analyte was evaluated at 5.00 (LQC), 300 (MQC) and 1500 (HQC) ng/mL. The matrix effect of the IS was determined at 1000 ng/mL. 

Recovery of SSa and SSd was determined at 5.00 (LQC), 300 (MQC) and 1500 (HQC) ng/mL, whereas for IS it was determined at 1000 g/mL. The specificity of the method was evaluated by comparing the chromatograms from six different blank rat plasma samples to evaluate the potential interferences at the retention times of analytes and IS. The sensitivity of the analyte was determined by calculating the signal-to-noise ratio of the lower limit of quantification (LLOQ) samples. Carryover of the analyte was determined by analysing blank plasma extract samples after injecting an upper limit of quantification (ULOQ) sample.

The calibration curve of SSa and SSd was obtained by plotting the peak area ratios of SSa and SSd to IS against the nominal concentration of calibration standards at the concentrations of 2.00–2000 ng/mL. Linearity was evaluated by weighted (1/×2) least-squares regression analysis. 

The intra- and inter-batch precision and accuracy were obtained by analysing three separate batches of rat plasma samples at two different concentrations. Each batch consisted of one set of calibration standards (eight concentration levels) and five replicates of QC samples at each of the LQC, MQC and HQC levels. Dilution integrity was performed to extend the upper concentration limit with acceptable precision and accuracy. Five replicates of the dilution QC samples at a concentration of about 10 times that of the ULOQ were diluted 10-fold with blank plasma. The diluted samples were processed and analysed. 

Stability experiments were performed to evaluate the analyte stability in stock solutions and in plasma samples under different conditions. Room temperature stability, refrigerated stability of extracted samples, freeze-thaw stability and long term stability were performed using three replicates at each QC level.

## 4. Conclusions

In this study, SSa-SSd-Lip was successfully prepared by the film dispersion technology. The statistical analysis of Plackett–Burman design, Box–Behnken design and desirability function enabled us to screen several significant process factors and to find out the optimum process conditions for SSa-SSd-Lip preparation in a quick and economical way. The optimum EPC/SSa-SSd ratio, the EPC/Chol ratio, the water temperature, pH of PBS and ultrasound time were 26.71, 4, 50 °C, 7.4 and 15 min, respectively. The entrapment efficiency of liposomes was markedly increased, the hemolysis was decreased, and the particle size was acceptable. The experimental results were in good agreement with the predicted values. In the pharmacokinetic study, the SSa-SSd-Lip increased the circulation time of SSa and SSd after intravenous administration. Encapsulation of SSa and SSd in liposomes significantly decreased the Cl (*p* < 0.05), and increased the AUC, MRT and T_1/2β_ (*p* < 0.05). The present study reveals that the SSa-SSd-Lip might provide an efficient approach to enhance the bioavailability of SSa and SSd and to reduce hemolysis so as to improve safety of the drugs. 
